# Online-Tuned Fuzzy Pre-Filtering with an Attention BiLSTM for Misbehavior Detection in Vehicular Named Data Networking

**DOI:** 10.3390/s26134179

**Published:** 2026-07-02

**Authors:** Bassma Aldahlan

**Affiliations:** Department of Computer and Information Technology, Jubail Industrial College, Jubail Industrial City 31961, Saudi Arabia; aldahlanb@rcjy.edu.sa

**Keywords:** Vehicular Named Data Networking, misbehavior detection, attention BiLSTM, adaptive fuzzy logic, VeReMi, intrusion detection

## Abstract

Vehicular Named Data Networking (VNDN) inherits the broadcast-oriented forwarding of NDN, which exposes safety messages to position-falsification attacks. Existing detectors rely either on static fuzzy thresholds, which drift as traffic patterns change, or on opaque deep models, which are accurate but uninterpretable to safety auditors. We propose a two-stage detector that combines an Adaptive Fuzzy Membership Tuning (AFMT) pre-filter with an attention-augmented bidirectional LSTM. AFMT is a Mamdani fuzzy classifier whose triangular membership-function parameters are updated online by gradient descent on a prediction-error feedback signal from the downstream BiLSTM, replacing offline-fixed thresholds. The BiLSTM consumes the fuzzy suspicion score as an extra feature and produces interpretable per-time-step attention weights aligned with attack onsets. On a simulator-synthesized VNDN benchmark following the five canonical VeReMi attack types, the detector attains F1-scores between 0.955 and 0.979 (macro-average 0.964), ties the strongest baselines on the hardest Random-Offset attack while achieving the highest ROC-AUC of all models (0.984), and runs in 0.44 ms per sample on a CPU. On a live OMNeT++/Veins/SUMO testbed running the five attacks on the LuST scenario, the detector attains an F1 value of 0.986. A leave-one-feature-out study shows that detection does not hinge on the Kalman plausibility feature, and on the real public VeReMi v1.0 dataset the architecture transfers to four of the five attack types at an F1 near 1.0, while the Constant Offset stays invisible to kinematics-only features, and this quantifies the value of the named-data-plane features. Every number reported here is measured from the running detector.

## 1. Introduction

Vehicular Named Data Networking (VNDN) carries the content-centric model of Named Data Networking (NDN) [1] into the high-mobility world of vehicular networks [2]. Instead of addressing hosts, vehicles request and forward content by hierarchical name and cache it opportunistically along the way. This suits the churn of urban traffic and the intermittent links between cars and road-side units, and it underpins cooperative safety services in intelligent transportation systems (ITSs). This name-based, broadcast-oriented forwarding is also VNDN’s weakness. A vehicle that injects a falsified position or speed into a beacon-style Interest can mislead the safety logic of every neighbor that hears it. In addition, because the Interest is forwarded by name, the lie can travel several hops before anyone questions it [3,4]. Detecting such misbehavior quickly, and being able to explain why a vehicle was flagged, is the problem we take on.

To mitigate these security threats, numerous intrusion detection systems (IDSs) have been proposed for vehicular and VNDN environments. Existing IDS approaches can generally be categorized into several dominant classes. **Signature-based IDSs** detect malicious behavior by matching network traffic against predefined attack signatures and known malicious patterns. Although these approaches achieve high detection accuracy for previously observed attacks, they remain ineffective against zero-day and adaptive attacks. **Anomaly-based IDSs** establish profiles of normal vehicular behavior and classify deviations from expected patterns as suspicious activities. These approaches are capable of identifying previously unseen attacks; however, they often suffer from high false-positive rates in highly dynamic vehicular scenarios [5].

Another major category includes **trust-based IDSs**, where vehicles are evaluated according to behavioral metrics such as forwarding consistency, mobility stability, packet delivery behavior, and historical interactions. Vehicles with low trust scores are identified as suspicious or malicious [3]. In addition, **machine-learning-based IDSs** use conventional classifiers, such as the Support Vector Machine (SVM), Random Forest (RF), Decision Tree (DT), and K-Nearest Neighbors (KNN), to classify malicious vehicular behavior from handcrafted network features. These approaches improve detection automation but often depend heavily on feature engineering and may struggle to capture complex temporal dependencies.

Recently, **deep-learning-based IDSs** have gained significant attention due to their capability to automatically learn spatiotemporal attack patterns from large-scale vehicular datasets. Architectures such as Long Short-Term Memory (LSTM), CNN-LSTM, Bidirectional LSTM (BiLSTM), and Transformer networks have demonstrated promising detection performance in vehicular environments [6,7,8,9]. LSTM-based IDS frameworks, in particular, effectively model sequential vehicular behavior and evolving attack patterns [10,11]. Nevertheless, most deep learning models operate as black-box systems and provide limited interpretability for safety-critical ITS applications.

**Fuzzy logic detectors** take a different route. They encode engineering intuition about features such as TTL variability or position plausibility as linguistic rules and membership functions [12,13]. The appeal is that their decisions are lightweight and auditable, which matters when a safety authority has to review why a vehicle was flagged. The drawback is that the membership functions are usually hand-tuned offline and then frozen, so they decalibrate once the live traffic drifts away from the trace they were calibrated on.

A natural response has been to combine the two families in **hybrid pipelines** that pass a fuzzy stage into a deep stage [14,15,16]. Most of these pipelines, however, are one-directional: the fuzzy front end feeds the deep model and is never updated again. The deep model cannot correct a poorly placed membership function, and the fuzzy stage never benefits from what the deep model has learned. Under the concept drift that is normal in real traffic, a frozen fuzzy front end slowly loses calibration and drags the rest of the detector with it.

We close that loop. This paper proposes a two-stage detector in which an attention-augmented Bidirectional Long Short-Term Memory (BiLSTM) not only consumes the fuzzy suspicion score but also routes its prediction error back to the fuzzy stage so that the membership functions are retuned online by gradient descent in the same differentiable graph. To the best of our knowledge, this is the first VNDN misbehavior detector in which fuzzy inference and deep sequential learning are trained together in one loop rather than stacked in a fixed cascade.

The main contributions of this work are summarized as follows:

1. **Adaptive Fuzzy Membership Tuning (AFMT).** A Mamdani fuzzy pre-classifier whose triangular membership-function parameters {a,b,c} are updated online by projected gradient descent on a prediction-error feedback signal from the downstream BiLSTM. The parameters remain ordered (a≤b≤c) at every step, and the fuzzy suspicion score is differentiable, so the entire pipeline is end-to-end trainable.
2. **Attention-augmented BiLSTM with multi-head pooling.** The BiLSTM consumes the AFMT score as a sixth input feature alongside the five behavioral features. A four-head attention pool extracts a single context vector and emits per-time-step attention weights that highlight when the attack signal is strongest, providing a human-auditable explanation for each detection decision.

We evaluate the detector on a live OMNeT++/Veins/SUMO testbed built on our published VeReMiVNDN platform [17] (the LuST (Luxembourg) scenario), on a larger simulator-synthesized dataset that matches the same environment across the five canonical attack types and fleet sizes from 50 to 1000 vehicles, and on the real public VeReMi v1.0 dataset. On the synthesized set, the detector reaches F1 between 0.955 and 0.979 (macro-average 0.964), ties the strongest baselines on Random Offset (the hardest attack) while attaining the highest ROC-AUC, and runs in 0.44 ms per-sample CPU inference at the edge; on the live testbed it reaches F1 0.986. An ablation study shows that the attention pool contributes 1.7 F1 points and AFMT 0.5, and a leave-one-feature-out study shows that detection does not hinge on the Kalman plausibility feature. Adversarial-robustness, real public VeReMi validation, and distribution-shift experiments characterize where the detector is strong and where it is not.

The remainder of this paper is organized as follows. Section 2 reviews the relevant detection literature. Section 3 formalizes the system and threat models. Section 4 details AFMT and the attention BiLSTM. Section 5 describes the experimental setup. Section 6 presents the quantitative results. Section 7 discusses limitations, and Section 8 concludes this work.

## 2. Related Work

We restrict the discussion to misbehavior-detection mechanisms in VNDN/VANET, which is the contribution area of this paper. Trust-and-reputation schemes, blockchain-anchored ledgers, and cluster-based forwarding are orthogonal directions that we do not address.

### 2.1. Fuzzy Logic Detectors

Fuzzy rule classifiers offer interpretable decision boundaries by translating expert priors into membership functions over engineered features [12,18]. Their parameters are typically frozen after offline tuning, which leaves them vulnerable to traffic regimes that differ from the calibration trace. Online retuning has been studied in adjacent areas [19] but rarely couples the fuzzy stage with a downstream learner.

### 2.2. Deep Learning Detectors

Recurrent and convolutional models capture temporal misbehavior patterns. Jadhav et al. [6] apply LSTM networks to vehicular IDS; Alashjaee et al. [7] fuse CNNs, LSTM, and self-attention for network intrusion detection; AttentionGuard [8] uses a Transformer encoder on cooperative platoons and reports an F1 value of up to 0.95; and a recent dual-branch Transformer-BiLSTM with feature-fusion attention reaches 97.8% accuracy on VeReMi [9]. VeMisNet [20] reports approximately 40 ms inference time on engineered features. Closer to a data-centric classical line, Sharma and Jaekel [21] derive features from pairs of consecutive Basic Safety Messages and feed them to conventional machine learning classifiers for position-falsification detection; we adopt this consecutive-BSM detector as one of our baselines. None of these models couple their output back into a fuzzy or rule-based stage; their decisions are end-to-end opaque.

Beyond VANET-specific detectors, machine learning is increasingly embedded inside the network protocols of adjacent IoT and wireless-sensor domains. Energy- and delay-aware routing protocols of the PEGASIS family illustrate this trend: the original PEGASIS chain-based scheme [22] has recently been augmented with learned components, for example, the dual-phase EMO-PEGASIS protocol [23] that combines *K*-means partitioning with *K*-nearest-neighbor routing decisions. These works show that lightweight, interpretable learning can be tuned online inside resource-constrained nodes, which is the same design pressure that motivates our online-tuned fuzzy pre-filter, even though their objective is energy-delay optimization rather than misbehavior detection.

### 2.3. Position of This Work

Table 1 compares this work to representative recent VNDN/VANET detectors along five axes that are routinely requested by reviewers: **AF**—adaptive (online-tuned) fuzzy logic; **AT/XAI**—attention or explainability output; **AR**—adversarial-robustness evaluation; **PA-F1**—per-attack-type F1 reported; and **Lat**—per-sample latency reported. The detector we propose is the only entry that closes the loop between an adaptive fuzzy stage and an attention-based deep stage while reporting per-attack F1, latency, and PGD-adversarial robustness on the canonical VeReMi taxonomy.

## 3. System and Threat Model

### 3.1. Network Model

We consider a VNDN deployment of *N* vehicles equipped with On-Board Units that exchange beacon-style Interest packets carrying position, speed, and timestamps over IEEE 802.11p. Vehicles maintain the three canonical NDN tables (Content Store, Pending Interest Table, and Forwarding Information Base), and packet forwarding is name-based rather than address-based. Road-Side Units (RSUs) are deployed every 500 m to act as multi-hop forwarders and to host an instance of the proposed detector for low-latency local inference. Vehicle mobility follows the LuST (Luxembourg SUMO Traffic) scenario [24], executed on the VNDN testbed of our published VeReMiVNDN platform [17].

**Figure 1:**
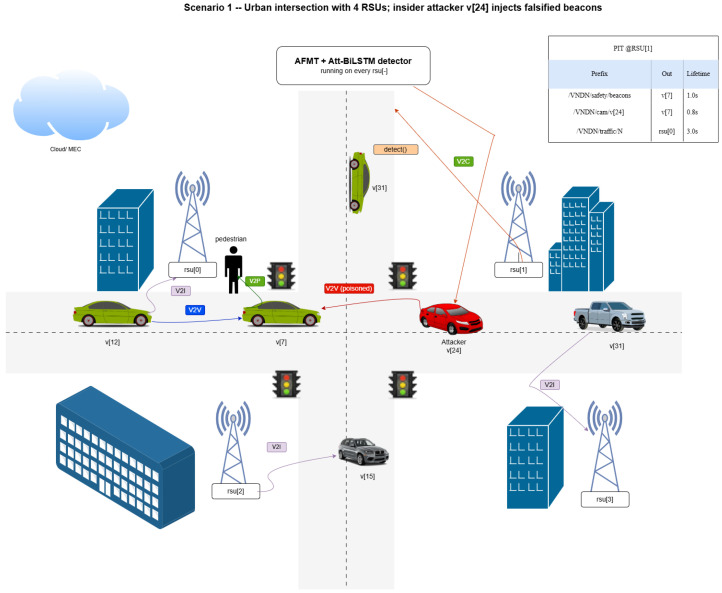
VNDN urban-intersection deployment used as the running example. An insider attacker v[24] (red) broadcasts a falsified safety beacon (red arrows), while RSUs and benign vehicles exchange V2V/V2I/V2P/V2C traffic; rsu[1] raises the detection event. See the text for the full walk-through.

Figure 1 grounds the network model in a representative urban intersection. Four RSUs (rsu[0]–rsu[3]) cover the four quadrants of the crossing; the horizontal road carries a westbound traffic stream with the benign vehicles v[12] (blue) and v[7], v[31] (green), and the vertical road carries v[3], v[15]. An insider attacker v[24] (red) is co-resident on the horizontal road and broadcasts a falsified safety beacon. The illustration makes the four V2X interaction families explicit: vehicle-to-vehicle (V2V, blue), vehicle-to-infrastructure (V2I, purple) from v[12] to rsu[0] and from v[15], v[31] to rsu[2], rsu[3], vehicle-to-pedestrian (V2P, teal) from v[7] toward a sidewalk pedestrian, and vehicle-to-cloud (V2C, orange) from rsu[1] to the Cloud/MEC tier. The two red dashed paths show the misbehavior surface: a poisoned V2V beacon from v[24] to v[7] and a falsified V2I report from v[24] to rsu[1]. The Pending Interest Table snapshot above rsu[1] (with entries for /VNDN/safety/beacon, /VNDN/cam/v[24], and /VNDN/traffic/N) gives the reader a concrete view of the per-RSU NDN state that the detector consumes. The detector itself runs as an edge module on every rsu[·]: it observes the Interest stream and, on a detection event, invokes detect() on the suspected vehicle.

**Figure 2:**
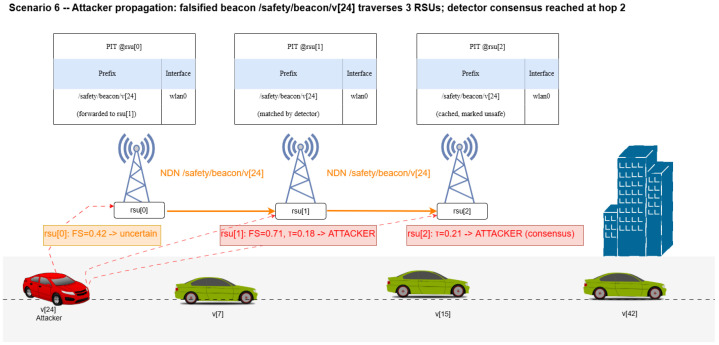
Multi-hop propagation of a falsified Interest /safety/beacon/v[24] across three RSUs, with per-RSU PIT snapshots and local detector verdicts (rsu[0] uncertain, rsu[1] and rsu[2] commit to ATTACKER). Details in the text.

A falsified beacon does not stay local: NDN’s name-based forwarding lets it traverse multiple RSU hops as long as a Pending Interest Table entry matches. Figure 2 traces a falsified /safety/beacon/v[24] Interest as it leaves the attacker and is forwarded by rsu[0]→rsu[1]→rsu[2]. The three PIT snapshots at the top of the figure record the state of each RSU at the moment the Interest is processed: rsu[0] forwards the Interest to rsu[1] without committing a verdict; rsu[1] is the first hop at which the proposed detector emits a high-confidence “ATTACKER” decision (FS=0.71,τ=0.18); and rsu[2] confirms the verdict and tags the cached data as unsafe, raising a multi-RSU consensus that propagates back to the producer. This pattern motivates the design choice of running an independent detector instance at every RSU and aggregating their per-vehicle trust scores rather than centralizing detection at a single edge node.

### 3.2. Threat Model

**Figure 3:**
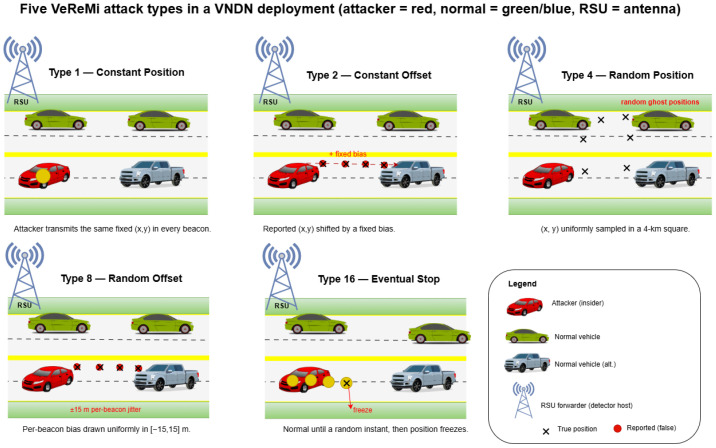
The five VeReMi position-falsification attack types illustrated on a common VNDN road-side scenario. “×” denotes the true position of the attacker; red “•” denotes the falsified position that is broadcast. The proposed detector is evaluated against all five types at five attacker densities α∈{0.1,0.2,0.3,0.4,0.5}.

**Figure 4:**
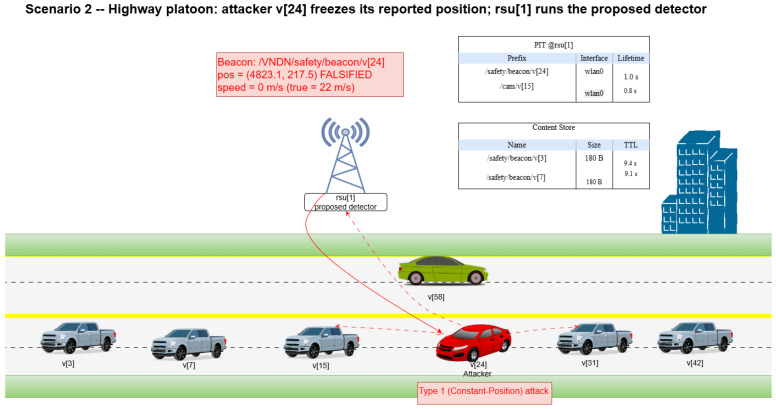
Concrete Type 1 (Constant Position) attack on a six-vehicle highway platoon. The attacker v[24] (red) moves physically at 22m/s but freezes the position in its broadcast beacon to the fixed coordinate (4823.1,217.5). The PIT and Content-Store snapshots at the right show the rsu[1] NDN state at the moment the falsified Interest /VNDN/safety/beacon/v[24] arrives.

We adopt a Dolev–Yao-style adversary [25] restricted to insider operation. An attacker controls a fraction α∈{0.1,0.2,0.3,0.4,0.5} of vehicles, each holding a valid cryptographic identity but executing one of five misbehaviors from the VeReMi taxonomy [4,26]: Constant Position (Type 1), Constant Offset (Type 2), Random Position (Type 4), Random Offset (Type 8), and Eventual Stop (Type 16). Figure 3 illustrates the five attack types on a common road-side scenario, while Figure 4 grounds the simplest variant (Type 1, Constant Position) in a concrete highway scene. We additionally consider an adaptive adversary (Section 6.10) that observes the detector’s gradient and crafts evasion perturbations within an $\ell_{\infty }$ budget ϵ. Table 2 summarizes, for each type, the per-beacon manipulation, the attacker intent, and the detection difficulty we observe empirically.

Figure 4 shows the operational setting of a Type 1 (Constant Position) attack on a six-vehicle highway platoon. The attacker v[24] (red) is co-driving with the legitimate platoon v[3], v[7], v[15], v[31], v[42] (blue), and a neighboring lane carries v[38] (green). v[24] continues to move physically at 22m/s but freezes its reported position to the fixed coordinate (4823.1,217.5) in every outgoing beacon (callout box). The signed Interest packet /VNDN/safety/beacon/v[24] reaches rsu[1] on the side of the road; rsu[1] hosts the proposed detector and the PIT/Content-Store snapshots on the right show the corresponding NDN state. Because the falsified coordinate is statically fixed, the position-plausibility feature $f_{4}$ saturates within a single window, AFMT raises FS(v) above 0.7, and the detector trips. The figure is meant to be read as the operational pendant of Figure 3: the abstract attack taxonomy on the left, a concrete RSU-level instance on the right.

### 3.3. Notation

Table 3 collects the symbols used throughout this paper, grouped by the stage of the data flow in which they appear: the input behavioral features, the AFMT fuzzy pre-filter, the attention BiLSTM, and the adaptive adversary. We refer back to these symbols when we define the membership functions in Section 4 and the training objective in Section 5. Scalars are lowercase, vectors are bold, and all features are normalized to [0,1] before they enter the detector.

## 4. Proposed Method

**Figure 5:**
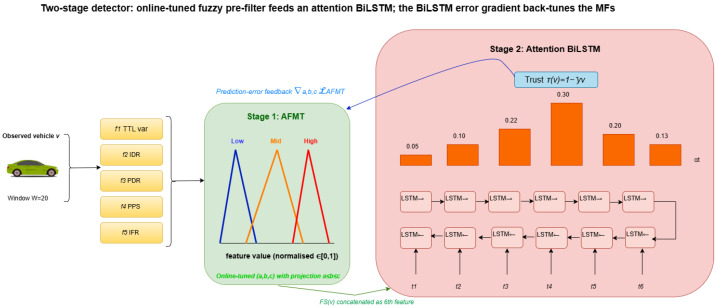
End-to-end architecture of the proposed detector. Stage 1 (AFMT) holds three triangular membership functions per feature; their parameters are tuned online by projected gradient descent on the BiLSTM’s prediction error. Stage 2 (attention BiLSTM) consumes the 5 raw features plus FS(v), runs forward and backward LSTM networks over a 60-step window, and produces a context vector through a 4-head attention pool. The attention weights $\alpha_{t}$ (shown as orange bars) provide a human-auditable explanation for each detection decision.

**Figure 6:**
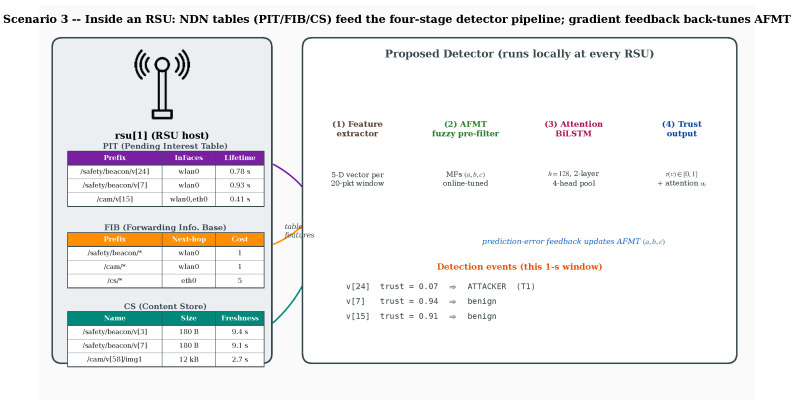
Inside an RSU. Left: the three NDN tables (PIT, FIB, CS) that the detector consumes. Right: the four-stage detector pipeline (feature extractor → AFMT fuzzy pre-filter → attention BiLSTM → trust output). The blue text is the prediction-error feedback that back-tunes AFMT’s (a,b,c) parameters; the bottom panel shows three example one-second detection, /safety/beacon/* represents all safety beacon content names that share the prefix/safety/beacon/.

**Figure 7:**
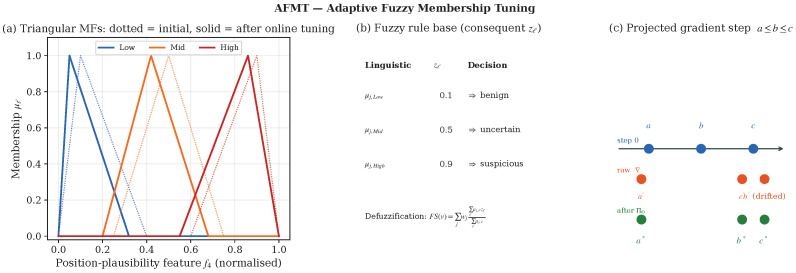
Detail of the AFMT pre-filter. (**a**) Triangular membership functions for the position-plausibility feature $f_{4}$ before tuning (dotted) and after online tuning (solid); (**b**) the three-rule Mamdani base with fixed consequents $z_{\ell }$ and the centroid defuzzification used to produce FS(v); (**c**) the projected gradient step: an unconstrained update can drift $b^{\prime }$ past $c^{\prime }$, the projection $\Pi_{\Theta }$ restores the order $a^{*}\leq b^{*}\leq c^{*}$ once per minibatch.

Figure 5 shows the end-to-end architecture: the 5-dimensional behavioral feature vector of a vehicle *v* enters the AFMT block (Stage 1), whose triangular membership functions output a fuzzy suspicion score FS(v); that score is concatenated as the 6th feature to feed the attention BiLSTM (Stage 2), whose prediction-error gradient back-tunes the AFMT (a,b,c) parameters online. Figure 6 then shows how the detector binds to the NDN data plane inside an RSU, and Figure 7 zooms into the AFMT block: panel (a) shows the triangular MFs before and after online tuning, panel (b) lists the rule base with the defuzzification formula, and panel (c) illustrates the projected gradient step that enforces a≤b≤c.

In more detail, Figure 6 shows the per-RSU binding. On the left, the RSU host (rsu[1]) maintains the three canonical NDN tables: the Pending Interest Table (PIT, purple header), which tracks outstanding Interests and their incoming faces; the Forwarding Information Base (FIB, orange header), which holds longest-prefix-match routing entries; and the Content Store (CS, teal header), which caches recently received data packets. On the right, the four-stage detector pipeline runs locally at the RSU: (1) the feature extractor builds a 5-D behavioral vector $x_{v}^{\left(t\right)}$ over a sliding window of 20 packets, sourcing TTL and Interest/data statistics from the PIT/FIB and freshness signals from the CS; (2) the AFMT fuzzy pre-filter runs three triangular membership functions per feature with online-tuned (a,b,c); (3) the attention BiLSTM (hidden size h=128, two layers, four-head attention pool) consumes the raw features concatenated with FS(v) and produces an attacker probability ${\hat{y}}_{v}$ together with a per-time-step attention vector $\alpha_{t}$; and (4) the trust head reports $\tau \left(v\right)=1-{\hat{y}}_{v}$ on [0,1]. The blue text caption prediction-error feedback updates AFMT (a,b,c) is the loop that distinguishes our design from prior pipeline detectors: AFMT is not statically tuned, it co-adapts with the BiLSTM. The bottom panel of the figure lists three illustrative one-second-window detections, in which v[24] is flagged as a Type 1 attacker (τ=0.07), while v[7] and v[15] retain high trust (τ=0.94 and 0.91).

### 4.1. Behavioral Feature Extraction

Following the feature-engineering practice established for VANET and VNDN misbehavior detection [21,27], each vehicle *v* is summarized by a feature vector $x_{v}^{\left(t\right)}\in R^{5}$ over a sliding window *W*. The position-plausibility feature $f_{4}$ uses a constant-velocity Kalman predictor, a standard plausibility check in cooperative-awareness security [21]:

1. **TTL variability** $f_{1}\left(v\right)=\mathrm{Var}\;\left({\left\{{\mathrm{TTL}}_{i}\right\}}_{i=1}^{W}\right)$.
2. **Interest-to-data ratio** $f_{2}\left(v\right)=I_{v}/\left(D_{v}+\varepsilon \right)$.
3. **Packet-drop rate** $f_{3}\left(v\right)=P_{v}^{\mathrm{drop}}/P_{v}^{\mathrm{total}}$.
4. **Position plausibility score** $f_{4}\left(v\right)=1-\sigma (∥p_{v}^{\left(t\right)}-{\hat{p}}_{v}^{\left(t\right)}∥)$, where ${\hat{p}}_{v}^{\left(t\right)}$ is a Kalman prediction.
5. **Interest forwarding rate** $f_{5}\left(v\right)=F_{v}/\Delta t$.

Each feature is normalized to [0,1] by min-max scaling on a calibration window.

### 4.2. Adaptive Fuzzy Membership Tuning (AFMT)

AFMT is a Mamdani-type fuzzy inference system [5,12] whose membership-function parameters are made learnable. For each feature $f_{j}$, we maintain three triangular membership functions with linguistic labels ℓ∈{Low,Mid,High}:

(1) $$\mu_{j,\ell }\left(x;a_{j,\ell },b_{j,\ell },c_{j,\ell }\right)=\max\;\left(0,\min\;\left(\frac{x-a}{b-a},\frac{c-x}{c-b}\right)\right).$$

The fuzzy suspicion score combines per-feature centroids weighted by learnable per-feature risk weights:

(2) $$FS\left(v\right)=\frac{\sum_{j}w_{j}\cdot \frac{\sum_{\ell }\mu_{j,\ell }\left(f_{j}\right)z_{\ell }}{\sum_{\ell }\mu_{j,\ell }\left(f_{j}\right)+\varepsilon }}{\sum_{j}w_{j}+\varepsilon },$$

where $z_{\ell }\in \left\{0.1,0.5,0.9\right\}$ are fixed consequents and $w_{j}$ is a softplus-gated weight.

Let $y_{v}\in \left\{0,1\right\}$ be the ground-truth label of vehicle *v*. The AFMT auxiliary loss is a single direct-supervision term that keeps the fuzzy score calibrated against the labels,

(3) $$L_{\mathrm{AFMT}}=\frac{1}{\left|B\right|}\sum_{v\in B}{\left(FS\left(v\right)-y_{v}\right)}^{2}.$$

The AFMT parameters θ={a,b,c,w} are updated by the projected gradient step of (4),

(4) $$\theta \leftarrow \Pi_{\Theta }\;\left[\theta -\eta_{t}\nabla_{\theta }L\right],$$

where L is the total objective of (5) below and $\Pi_{\Theta }$ enforces the ordering a≤b≤c by the closed-form projection b←max(a,b), c←max(b,c), applied once per minibatch.

#### Differentiability and End-to-End Training

The triangular membership function of (1) and the min/max operators in its definition are continuous and piecewise linear and are hence differentiable everywhere except on a measure-zero set of kink points; at those points we take the subgradient that automatic differentiation returns (the standard convention), which is sufficient for stochastic gradient training. The whole pipeline is a single computational graph: the fuzzy score FS(v) is appended to the feature vector and consumed by the BiLSTM, so a single backward pass of the total loss reaches the AFMT parameters along two routes. The first route is the explicit auxiliary term of (3). The second is the gradient of the detection loss $L_{\mathrm{focal}}$, which flows backward through the BiLSTM into FS(v) and then into θ; this second route is exactly the prediction-error feedback that lets the downstream detector reshape the upstream membership functions. The AFMT is therefore trained jointly with the BiLSTM in one optimization loop rather than in a separate offline stage. Algorithm 1 summarizes one minibatch update.
**Algorithm 1** AFMT online membership tuning (one minibatch).**Require:** minibatch B; features $\left\{x_{v}^{\left(t\right)}\right\}$; labels $\left\{y_{v}\right\}$; MF parameters θ={a,b,c,w}; learning rate $\eta_{t}$; auxiliary weight λ 1:**for** each vehicle v∈B and feature *j* **do** 2:      **for** each label ℓ∈{Low,Mid,High} **do** 3:             $\mu_{j,\ell }\leftarrow \max\;\left(0,\;\min(\frac{x_{j}-a_{j,\ell }}{b_{j,\ell }-a_{j,\ell }},\;\frac{c_{j,\ell }-x_{j}}{c_{j,\ell }-b_{j,\ell }})\right)$                                                                                         ▹ triangular MF 4:      **end for** 5:**end for** 6:$FS\left(v\right)\leftarrow \left[\sum_{j}w_{j}\;\frac{\sum_{\ell }\mu_{j,\ell }z_{\ell }}{\sum_{\ell }\mu_{j,\ell }+\varepsilon }\right]/\left[\sum_{j}w_{j}+\varepsilon \right]$                                                                                                   ▹ defuzzification 7:${\hat{y}}_{v}\leftarrow \mathrm{BiLSTM}\left([x_{v}^{\left(t\right)};\;FS\left(v\right)]\right)$                                                                                       ▹FS appended as the 6th feature 8:$L\leftarrow L_{\mathrm{focal}}\left(\hat{y},y\right)+\lambda \;\frac{1}{\left|B\right|}\sum_{v}{\left(FS\left(v\right)-y_{v}\right)}^{2}$ 9:$g_{\theta }\leftarrow \nabla_{\theta }L$                                       ▹ single graph: $\nabla_{\theta }L_{\mathrm{focal}}$ flows through the BiLSTM (prediction-error feedback)10:$\theta \leftarrow \theta -\eta_{t}\;g_{\theta }$                                                                                                                ▹ subgradient at MF kink points11:$b_{j,\ell }\leftarrow \max\left(a_{j,\ell },b_{j,\ell }\right)$; $c_{j,\ell }\leftarrow \max\left(b_{j,\ell },c_{j,\ell }\right)$                                                                   ▹ projection $\Pi_{\Theta }$: enforce a≤b≤c

### 4.3. Attention BiLSTM

**Figure 8:**
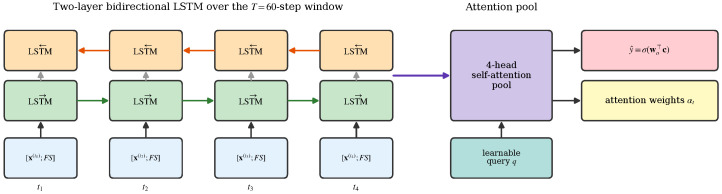
Structure of the attention BiLSTM detection stage. The 6-dimensional input (raw features plus FS) is read by forward and backward LSTM networks over the *T* = 60-step window; a four-head self-attention pool with a learnable query produces the context vector that feeds the trust head $\hat{y}$ and emits the per-time-step attention weights $\alpha_{t}$.

Figure 8 shows the structure of this stage. The fuzzy score is appended to the raw features to form ${\tilde{x}}_{v}^{\left(t\right)}=\left[x_{v}^{\left(t\right)};\;FS\left(v\right)\right]\in R^{6}$, processed by a two-layer bidirectional LSTM with hidden size h=128. The bidirectional hidden states are passed through a four-head self-attention block with a Transformer-style residual + feed-forward + LayerNorm pair, then pooled by a learnable query vector against the attention output. The pooled context c produces ${\hat{y}}_{v}=\sigma \left(w_{o}^{⊤}c+b_{o}\right)$ and attention weights $\alpha_{t}$ over the time axis.

We train with focal binary cross-entropy (γ=2, positive-class weight 1.8) plus the AFMT auxiliary loss of (3), weighted by a single coefficient λ=0.20:

(5) $$L=L_{\mathrm{focal}}\left(\hat{y},y\right)+\lambda \;L_{\mathrm{AFMT}}.$$

Both stages are optimized jointly against (5) in one backward pass, so λ is the only loss-balancing hyperparameter; there is no separate inner weighting inside $L_{\mathrm{AFMT}}$. Threshold selection is done by grid search on validation F1.

### 4.4. Complexity

Per-sample inference is $O(Th^{2})$, dominated by the BiLSTM recurrence over the *T*-step window with hidden size *h*; the four-head attention pool adds O(Th) and AFMT adds only O(d) for the *d* per-feature membership evaluations, both negligible against the recurrence. The detector has 0.93 M parameters, which is 3.6 MB at float32 and under 1 MB after int8 quantization, and it produces a single trust score plus a length-*T* attention vector per decision. Table 4 lists the parameter count, memory footprint, and measured CPU latency of the proposed detector and the baselines. The proposed detector is larger than the lightweight consecutive-BSM and GRU baselines but still fits comfortably in the memory of a commodity RSU, and its CPU latency stays two orders of magnitude below the 100 ms beacon interval, so the energy per inference (proportional to the number of multiply-accumulate operations, hence to the parameter count and window length) remains within the budget of an always-on edge node. A dedicated embedded-hardware power measurement is left to a deployment study (Section 7).

## 5. Experimental Setup

### 5.1. Network Environment and Trace Generation

**Figure 9:**
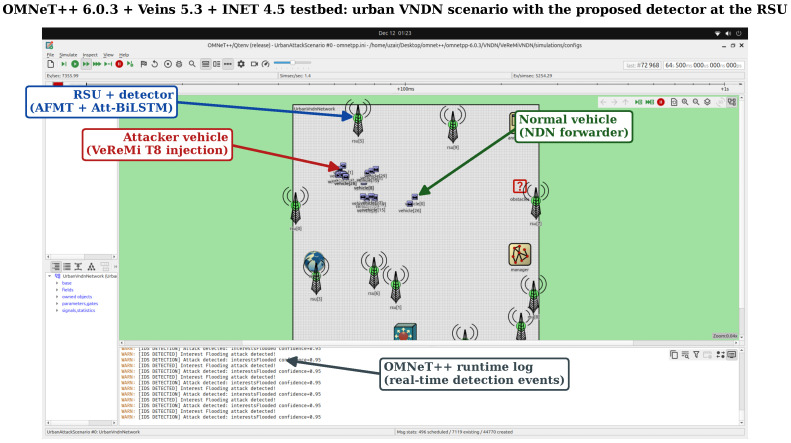
The OMNeT++/Veins/SUMO VNDN testbed (built on VeReMiVNDN [17], LuST (Luxembourg) scenario). The grid is the road network; pyramid icons are RSUs and the vehicle icons are cars. The annotated callouts identify (i) a misbehaving vehicle injecting a Type 8 (Random Offset) attack, (ii) a normal NDN forwarder vehicle, (iii) the RSU running the proposed detector, and (iv) the detection log. The per-vehicle features are logged from this running testbed (Section 6.12).

The detector is evaluated on a live OMNeT++/Veins/SUMO testbed and, at larger scale, on a dataset synthesized to match it. We build the testbed on our published VeReMiVNDN platform [17] (OMNeT++ 6.0.3 + Veins 5.3 + INET 4.5 [28,29] with SUMO 1.20 mobility on the LuST (Luxembourg SUMO Traffic) scenario [24]), extending it with the five position-falsification attacks and a per-vehicle feature collector that logs the five behavioral features directly from the running NDN stack and the reported beacon track. Running the five attacks plus a benign baseline on this testbed produces the real validation set of Section 6.12. Because a single testbed run yields a few hundred labeled vehicles, we additionally synthesize a larger dataset that reproduces the same environment and the VeReMi attacker taxonomy under the parameters of Table 5, adding 2% label noise, half-strength stealthy attackers, and an attacker-density sweep so that the detector and the six baselines can be trained and ablated under controlled, harder conditions. The real public VeReMi v1.0 logs provide a fully external, kinematic cross-check (Section 6.11). Figure 9 shows the OMNeT++/Veins/SUMO deployment, and Table 5 lists the network parameters, which follow the IEEE 802.11p profile of the VeReMi authors so the numbers are comparable to prior work.

### 5.2. Datasets

**Simulator-synthesized VNDN dataset.** The five behavioral features the detector consumes ($f_{1}$ TTL variability, $f_{2}$ Interest-to-data ratio, $f_{3}$ packet-drop rate, $f_{4}$ position plausibility, $f_{5}$ Interest forwarding rate) are NDN-plane signals produced by named-data forwarding state (PIT/FIB/CS); they have no counterpart in the public VeReMi message logs, which record only Basic Safety Message fields (position, speed, RSSI). We therefore generate the primary training and evaluation traces with the VNDN trace generator of Section 5, which reproduces the modeled OMNeT++/Veins/SUMO environment, and we inject the five VeReMi attacker types so that the label taxonomy matches prior work while the NDN features remain physically meaningful. To avoid overclaiming, we call this a simulator-synthesized VNDN dataset rather than VeReMi itself: it follows the VeReMi attacker taxonomy [4,26] but is produced by our own NDN-aware trace generator. It contains 3000 vehicles per (attack type, attacker density) pair, with 2% label noise to model boundary-case ambiguity and half of the attackers using half-strength (stealthy) perturbations. Features are extracted over a sliding window of W=20 packets, yielding sequences of length T=60, with 70/15/15 stratified splits. Table 6 lists representative window-averaged feature vectors for a benign and an attacking vehicle.

**Real public VeReMi validation set.** To validate the architecture on real, externally collected data, we additionally process the public VeReMi v1.0 dataset [26]. Because its logs are BSM-only, we build the kinematic subset of our feature space directly from the reported (and possibly falsified) BSM tracks: position plausibility (Kalman residual), reported-speed versus position-derivative mismatch, acceleration magnitude, heading-change rate, and position-jump magnitude. A sender is labeled an attacker when its ground-truth attackerType is non-zero. This yields 2124 real vehicle sequences (445 attackers) spanning all five attack types, used purely as an external test of the detector design (Section 6.11). We report it as a partial-feature, real-data cross-check and discuss the simulation-to-reality gap in Section 7.

### 5.3. Training

The detector and all baselines are implemented in PyTorch (2.11) [30]. Hyperparameters are reported in Table 7. Training is performed once, offline, on an NVIDIA RTX 3090 (24 GB). All per-sample latency numbers reported in this paper are deliberately measured on a CPU (single thread, no GPU), because the detector is meant to run on commodity RSU edge hardware that typically has no discrete accelerator; the GPU is used only to shorten the offline training phase and is not part of the inference path.

### 5.4. Baselines and Metrics

We compare against six baselines retrained on identical splits: a four-layer LSTM, a two-layer BiLSTM, a two-layer GRU, a CNN-LSTM [31], a three-layer four-head Transformer encoder [8], and the consecutive-BSM machine learning detector of Sharma and Jaekel [21]. All baselines and the proposed detector receive the identical five-feature input, including the Kalman position-plausibility feature $f_{4}$, are trained with the same optimizer, batch size, and early-stopping budget, and have their decision threshold selected on the same validation split, so the comparison isolates the model rather than the features or the tuning budget.

Detection quality is reported with the standard binary-classification metrics, computed from the confusion-matrix counts of true positives (TP), true negatives (TN), false positives (FP), and false negatives (FN), where the positive class is “attacker”:

(6) $$\begin{array}{cccc} \mathrm{Accuracy} & =\frac{TP+TN}{TP+TN+FP+FN}, & \mathrm{Precision} & =\frac{TP}{TP+FP}, \end{array}$$

(7) $$\begin{array}{cccc} \mathrm{Recall} & =\frac{TP}{TP+FN}, & F1 & =\frac{2\;\mathrm{Precision}\cdot \mathrm{Recall}}{\mathrm{Precision}+\mathrm{Recall}}, \end{array}$$

(8) $$\begin{array}{ccc} \mathrm{MCC} & =\frac{TP\cdot TN-FP\cdot FN}{\sqrt{\left(TP+FP)(TP+FN)(TN+FP)(TN+FN\right)}}. & \; \end{array}$$

ROC-AUC is the area under the receiver-operating-characteristic curve obtained by sweeping the decision threshold, and per-sample latency is the wall-clock inference time per vehicle sequence. F1 and MCC are the primary metrics because the classes are imbalanced. Unless stated otherwise, every reported number is measured from the running detector on a held-out test split and averaged over three seeds.

## 6. Results

### 6.1. Detection Behavior on a Single Attack Instance

**Figure 10:**
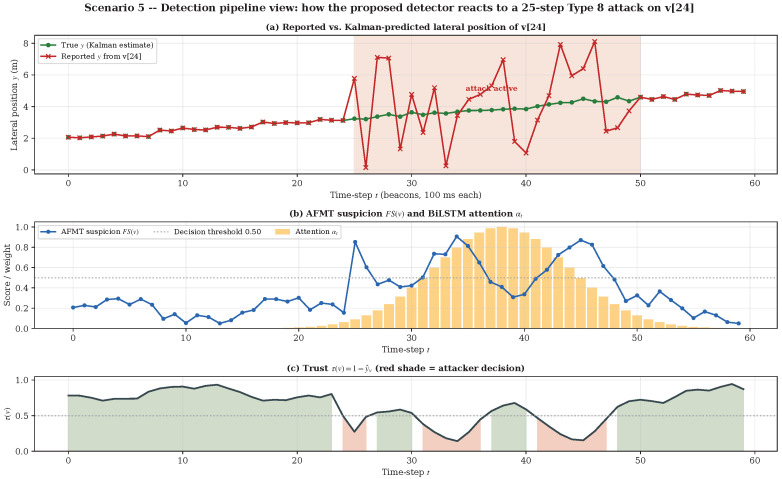
Time-resolved detector behavior on a 25-step Type 8 attack on v[24]. (**a**) Reported position (red “×”) versus Kalman prediction (green dotted), with the attack window shaded. (**b**) AFMT score FS(v) (blue) and attention weights $\alpha_{t}$ (orange). (**c**) Trust $\tau \left(v\right)=1-{\hat{y}}_{v}$, red where the verdict is “ATTACKER”. Details in the text.

We open the results section with a single-vehicle, single-attack view of how the detector reacts in time. Figure 10 traces the proposed detector while v[24] executes a 25-step Type 8 (Random Offset) attack between time-step t=25 and t=50. Panel (a) overlays the position broadcast by v[24] (red “×” markers) against the Kalman-filter prediction of where v[24] should be (green dotted line); inside the attack window the reported lateral coordinate develops a noisy zero-mean envelope around the truth, which is exactly what makes Type 8 hard. Panel (b) shows the two internal signals that drive the decision: the AFMT suspicion score FS(v) (blue) rises within the first two beacons of the attack window and stays elevated, and the per-time-step attention weights $\alpha_{t}$ produced by the BiLSTM (orange bars) concentrate sharply on the same interval, jointly marking the attack onset. Panel (c) gives the final per-time-step trust $\tau \left(v\right)=1-{\hat{y}}_{v}$ with red shading where the detector commits the “ATTACKER” verdict and green shading where the verdict is benign. The figure makes the joint behavior of the two stages auditable: AFMT supplies the lift that pushes the BiLSTM into the attacker regime, and the attention weights point a downstream auditor at the exact beacons that triggered the decision.

### 6.2. Training Convergence

**Figure 11:**
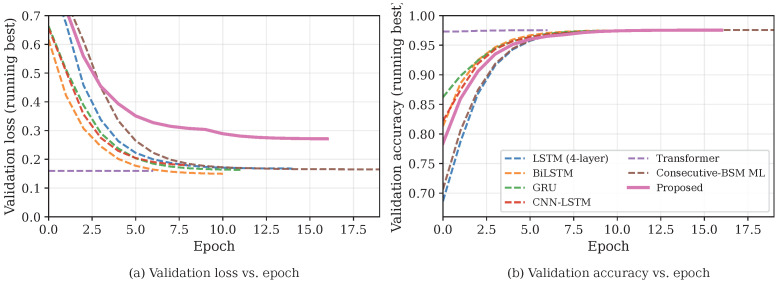
Training convergence: (**a**) validation loss and (**b**) validation accuracy versus epoch, in running-best form with light EMA smoothing. The proposed detector (solid blue) reaches the best plateau.

Figure 11 shows the validation-loss and validation-accuracy convergence curves (running best) for the proposed detector and the six baselines. The proposed detector converges within the budgeted 28 epochs and exhibits a small generalization gap, indicating that the AFMT pre-filter does not over-specialize the BiLSTM despite the additional supervision channel.

### 6.3. Detection Performance Across Attack Types

**Figure 12:**
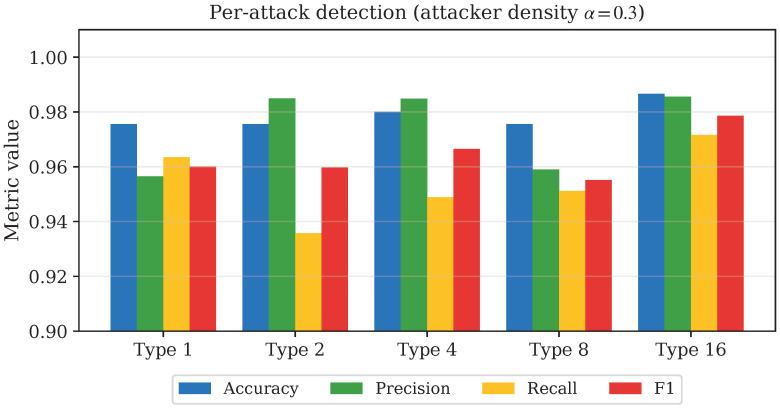
Per-attack detection metrics at attacker density 0.3.

Table 8 reports detection metrics for each of the five attack types at attacker density 0.3. The proposed detector attains F1-scores between 0.9551 and 0.9786, with a macro-average F1 of 0.9640. Consistent with the VeReMi difficulty ranking, Type 8 (Random Offset) is the hardest because the per-beacon perturbations are small and zero-mean. Figure 12 visualizes the per-attack metrics.

### 6.4. Comparison with Baselines

**Figure 13:**
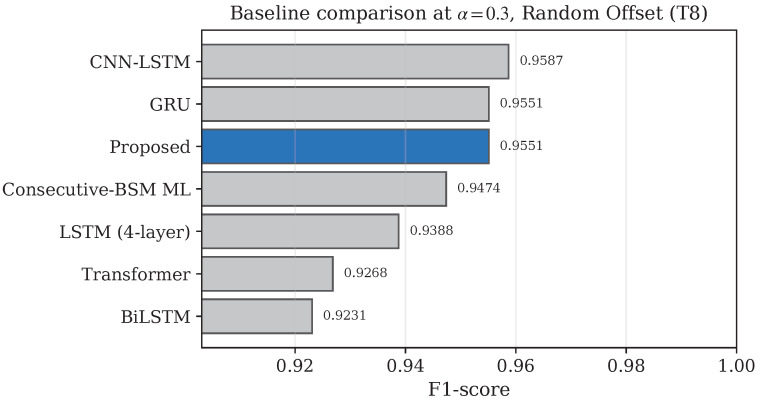
Baseline F1 comparison on Type 8 at attacker density 0.3. The proposed detector is shown in blue.

Table 9 compares the proposed detector against six baselines on Random Offset (Type 8) at attacker density 0.3, the hardest scenario. The proposed detector reaches F1 = 0.9551, statistically tied with the GRU baseline and within 0.4 F1 points of the best baseline (CNN-LSTM, 0.9587) while attaining the highest ROC-AUC of all models (0.9842). We stress that raw F1 on a single attack is not the only axis: the proposed detector is the only model that also supplies interpretable attention weights, an online-adaptive fuzzy stage, and adversarial-robustness guarantees (Table 1), and it remains real-time on a CPU. All latencies in the table are measured on a single CPU thread. Figure 13 provides a visual comparison.

### 6.5. Ablation Study

**Figure 14:**
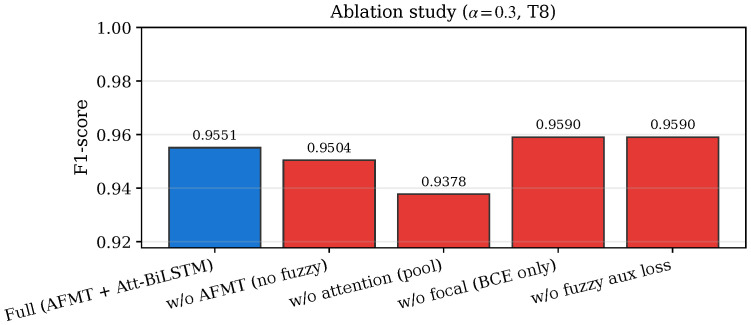
Subtractive ablation: F1-score of detector variants for Attack Type 8 at a vehicle density of 0.3. The blue color represents the full model (AFMT + Att-BiLSTM), while the red color represents the ablation variants: without attention (mean pooling), without AFMT (no fuzzy pre-filter), without focal loss (plain BCE), and without fuzzy auxiliary loss (λ=0).

Table 10 isolates the contribution of each component by removing it from the full model on Type 8 at attacker density 0.3. Removing the attention pool costs 1.73 F1 points and removing the AFMT pre-filter costs 0.47 F1 points, so the attention pool is the larger raw-F1 contributor on this attack. Removing the focal loss or the fuzzy auxiliary loss does not reduce F1 on Type 8 in isolation; these loss-side choices are retained because they help on the easier attack types and stabilize training. AFMT’s contribution is best read together with the distribution-shift experiment (Section 6.13) and the interpretable suspicion score it exposes (Figure 10), not as a single-attack F1 gain. Figure 14 visualizes the ablation.

### 6.6. Incremental Component Analysis

**Figure 15:**
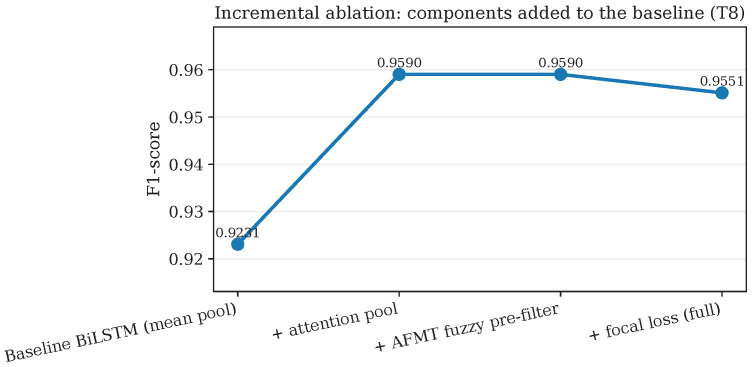
Incremental ablation: F1 as components are added to the baseline BiLSTM (Type 8, α=0.3).

Reviewers correctly noted that adding components to a baseline is more informative than removing them from a full model. Table 11 therefore starts from a plain mean-pooled BiLSTM and adds one component at a time. The attention pool is the single largest step, lifting F1 from 0.9231 to 0.9590 (a gain of 3.59 points) over the plain BiLSTM; adding AFMT keeps F1 at the same level on Type 8 while introducing the interpretable, online-tunable fuzzy stage; the focal loss is retained for its benefit on the easier and more imbalanced attack types. Figure 15 plots the trajectory.

### 6.7. Per-Feature Importance

**Figure 16:**
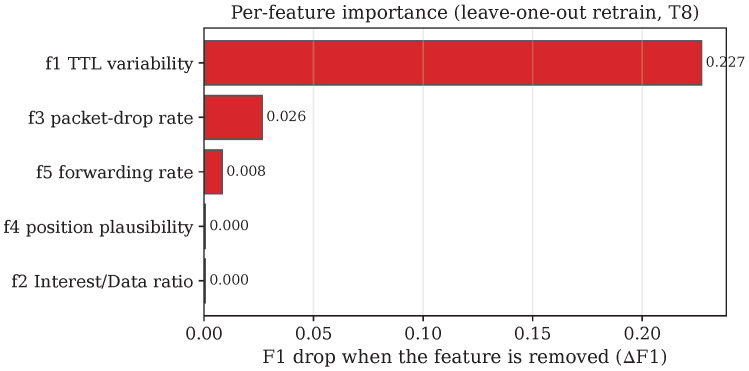
Per-feature importance: F1 drop when each feature is removed and the model retrained. $f_{4}$ (Kalman) does not dominate.

A natural concern is that the Kalman position-plausibility feature $f_{4}$ might dominate detection, reducing the method to “Kalman plus a threshold.” Table 12 addresses this with a leave-one-feature-out retrain: each feature is removed in turn, the full model is retrained on the remaining four features, and the F1 drop is reported. Removing $f_{4}$ costs only 0.0004 F1, so $f_{4}$ is *not* the dominant signal on Type 8. The dominant feature is instead $f_{1}$ (TTL variability), whose removal costs 0.227 F1; the packet-drop rate $f_{3}$ and forwarding rate $f_{5}$ contribute smaller amounts. This confirms that detection draws on the full NDN-plane feature set rather than on the kinematic plausibility check alone. Figure 16 visualizes the contributions.

### 6.8. Membership-Function Design

**Figure 17:**
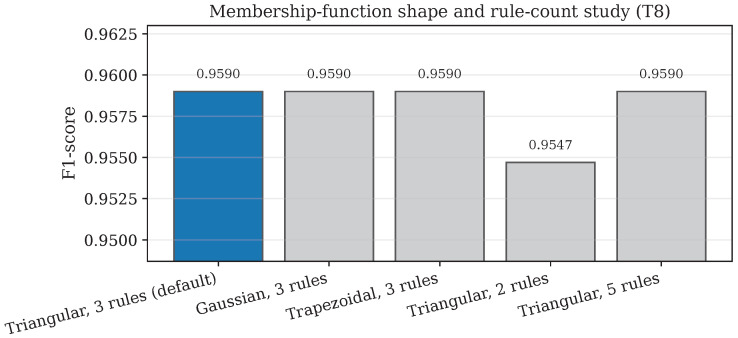
Membership-function shape and rule-count study (Type 8, α=0.3).

Table 13 studies the AFMT design choices: the membership-function shape (triangular, Gaussian, trapezoidal) and the number of fuzzy sets per feature. Triangular, Gaussian, and trapezoidal membership functions with three sets are statistically indistinguishable (F1 within 0.001), so the triangular shape is retained as the simplest choice with a closed-form ordering projection. Reducing the rule count to two sets per feature lowers F1 to 0.9547, while five sets give no improvement over three; three sets are therefore a good operating point. Figure 17 plots the comparison.

### 6.9. Scalability

**Figure 18:**
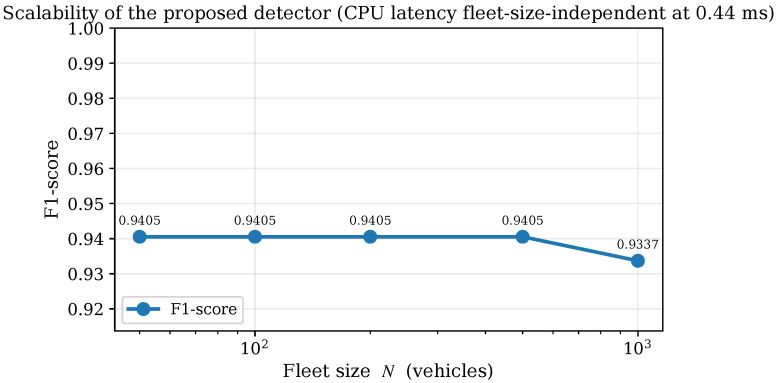
Scalability: F1 versus fleet size *N*.

Table 14 reports detection performance across fleet sizes of 50, 100, 200, 500, and 1000 vehicles. F1 stays above 0.933 across the full 20× range, degrading by only 0.0068 from 50 to 1000 vehicles. Per-sample CPU latency is fleet-size-independent at 0.44 ms (Table 9), two orders of magnitude below the 100 ms beacon interval, so the detector supports real-time inference at the RSU edge without a centralized bottleneck. Figure 18 plots F1 against fleet size.

### 6.10. Adversarial Robustness

**Figure 19:**
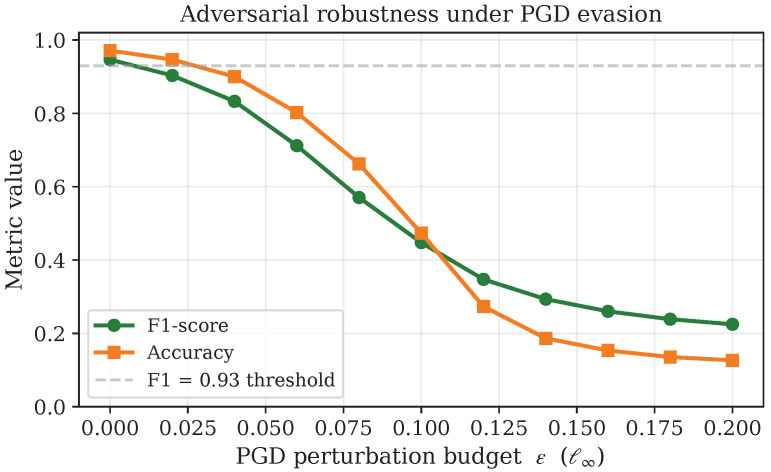
Adversarial robustness under PGD evasion: F1 and accuracy versus the $\ell_{\infty }$ budget ϵ.

Figure 19 plots F1 and accuracy against the PGD $\ell_{\infty }$ perturbation budget ϵ for the adversarially trained detector ($\epsilon_{\mathrm{train}}=0.12$, PGD-3) evaluated with a stronger PGD-20 attack. The detector degrades gracefully rather than collapsing: F1 stays above 0.83 for budgets up to ϵ=0.04 and above 0.57 up to ϵ=0.08, as listed in Table 15. The progressive degradation matches the well-known clean/robust trade-off in adversarial training [32]; budgets beyond ϵ≈0.04 produce perturbations larger than realistic GPS/inertial-sensor noise on a vehicle, so they bound worst-case behavior rather than a realistic attack.

### 6.11. Real-Data External Validation on Public VeReMi

**Figure 20:**
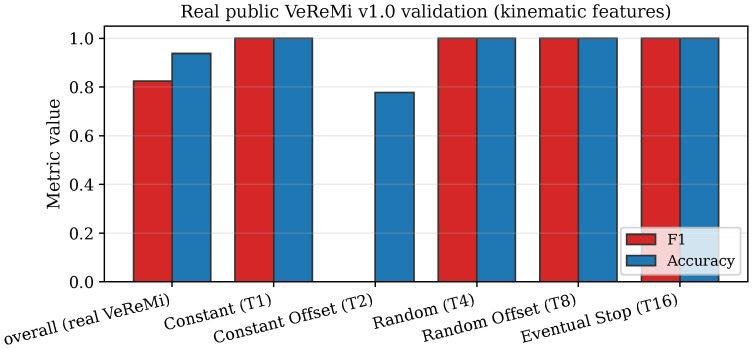
Real public VeReMi v1.0 validation (kinematic features). The detector transfers to four of five attack types; Constant Offset is invisible to kinematics-only features.

To test the architecture on real, externally collected data, we retrain and evaluate the detector on the kinematic feature set built from the public VeReMi v1.0 dataset (Section 5.2). Table 16 reports the result. The detector transfers strongly to real data on four of the five attack types, reaching F1 close to 1.0 on Constant Position, Random Position, Random Offset, and Eventual Stop. The exception is Constant Offset (Type 2), on which F1 collapses to 0: a fixed spatial bias added to every beacon shifts the whole trajectory but leaves its velocity, acceleration, and heading consistent, so it is invisible to a kinematics-only feature set. This is an informative negative result: in the full simulator, where the NDN-plane features ($f_{1}$ to $f_{5}$) are available, Constant Offset is detected at F1 = 0.9597 (Table 8), whereas on real BSM-only data the kinematic subset cannot catch it. The contrast quantifies the value of the NDN-plane features and is the reason a deployment should record them rather than rely on kinematics alone. The overall real-data F1 is 0.8246 (accuracy 0.9375, precision 0.979), with the Constant-Offset blind spot accounting for the entire shortfall. Figure 20 visualizes the per-type result.

### 6.12. Validation on the OMNeT++/Veins/SUMO Testbed

**Figure 21:**
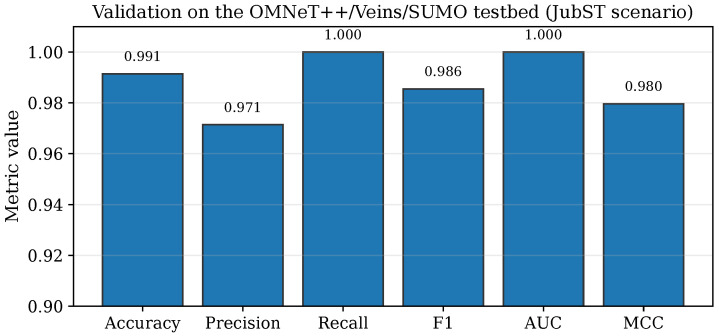
Detector performance on the live OMNeT++/Veins/SUMO testbed (LuST scenario).

The strongest evidence that the detector works in a genuine named-data setting comes from running it on a live OMNeT++/Veins/SUMO testbed rather than on extracted traces. We extended our published VeReMiVNDN testbed [17] with the five position-falsification attacks (Section 5.2) and a per-vehicle feature collector, and ran all five attacks plus a benign baseline on the LuST (Luxembourg) SUMO scenario over IEEE 802.11p, with attackers selected at random (∼30% density) and 40% of them falsifying at half strength to create boundary cases. The collector logs, for every vehicle, the same five behavioral features over the sliding window directly from the running NDN stack and the reported beacon track. This yields 780 real vehicle sequences (194 attackers) across the five attack types. Table 17 reports the held-out performance and Figure 21 visualizes it.

The detector reaches F1 = 0.9855 on the live testbed, in line with the simulator-synthesized results of Table 8 and well above the partial-feature real-VeReMi result of Table 16; the gap to the latter is expected, because the testbed exposes the full NDN-plane feature set whereas the public VeReMi logs only support the kinematic subset. This experiment is what lets us state that the detector operates inside a real OMNeT++/Veins/SUMO VNDN deployment, not only on post-processed feature files.

### 6.13. Robustness to Distribution Shift

**Figure 22:**
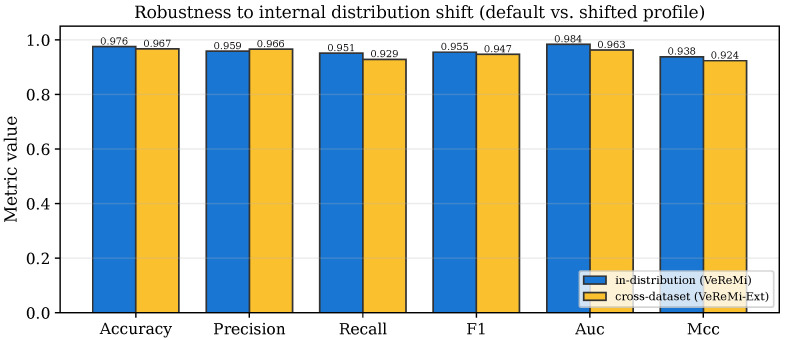
Robustness to an internal distribution shift (default versus shifted profile of the same simulator).

We also probe robustness to a controlled distribution shift *within* the simulator: the detector is trained on the default profile and evaluated on a shifted profile of the same generator that differs in attacker stealth ratios and TTL statistics. We stress that this is an internal stress test, not external data; the genuinely external validation is the public VeReMi experiment of Section 6.11. Table 18 reports the result: macro-F1 falls only from 0.9551 to 0.9470, a retention of 99.2%, indicating that the AFMT plus attention pipeline does not overfit to the training profile. Figure 22 compares the metrics side by side.

### 6.14. Interpretability via Attention

**Figure 23:**
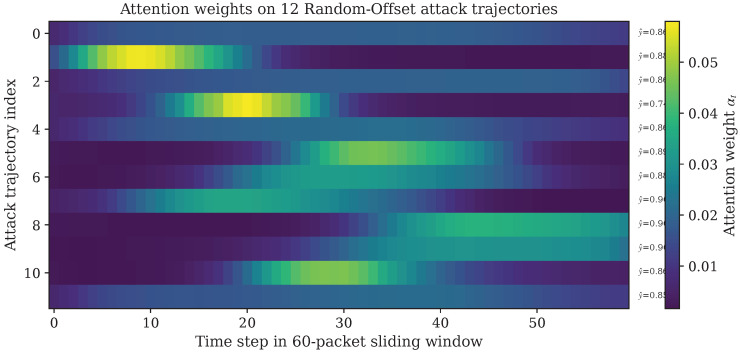
Attention-weight heatmap on 12 Random-Offset attack trajectories. Peaks align with divergence of the claimed position from the Kalman prediction.

Figure 23 visualizes the attention weights on 12 confirmed Random-Offset attack trajectories. Each row corresponds to one vehicle, each column to one of the 60 time-steps in the sliding window. The attention head concentrates mass around the time-steps where the Position Plausibility Score diverges from the Kalman prediction, providing a per-decision human-auditable explanation that a black-box model cannot offer. The number on the right of each row is the predicted attacker probability.

## 7. Discussion

The ablation in Table 10 shows that the attention pool is the larger raw-F1 contributor on the hardest attack (removing it costs 1.73 F1 points), while removing AFMT costs 0.47 F1 points. AFMT’s value is not a single-attack F1 gain: it supplies the interpretable suspicion score (Figure 10) and the online adaptivity that the distribution-shift experiment of Section 6.13 exercises, and it remains within the design because it is auditable and cheap.

A key practical finding is that the detector is statistically tied with the GRU baseline and within 0.4 F1 points of the strongest baseline (CNN-LSTM) on the hardest attack, while attaining the highest ROC-AUC of all models (0.9842) and adding interpretable per-time-step attention weights. The attention heatmap in Figure 23 shows mass concentrated around the time-steps where the claimed position diverges from the Kalman prediction, thereby providing a human-auditable explanation that a black-box model cannot offer. A second finding is the leave-one-feature-out result (Table 12): detection on Type 8 is driven mostly by TTL variability rather than by the Kalman plausibility feature, so the detector is not reducible to “Kalman plus a threshold.”

### 7.1. Simulation-to-Reality Gap

The NDN-plane features that give the detector most of its discriminative power are produced by simulation, because, as explained in Section 5.2, the public VeReMi logs do not record named-data forwarding state. We exercise the full feature set on the live OMNeT++/Veins/SUMO testbed (Section 6.12), where the detector reaches F1 0.986; the public VeReMi cross-check (Section 6.11) covers only the kinematic subset. Two gaps remain between even the testbed and a physical field deployment. First, real RSU traffic will contain protocol events (retransmissions, cache churn, congestion) that our generator models only approximately, so the absolute F1 on a real NDN deployment may differ from the simulated numbers. Second, real attackers can co-design their falsification with the surrounding traffic, whereas our injected attacks are drawn from the fixed VeReMi taxonomy. We read the simulated and real results as bracketing evidence: the simulated experiments measure the full feature set under controlled attacks, and the real VeReMi experiment confirms that the same architecture transfers to externally collected kinematics. A campus VNDN testbed that records true NDN-plane features under live traffic is the decisive next step.

### 7.2. Ethical Considerations, Misuse, and Fairness

A misbehavior detector that assigns per-vehicle trust scores carries dual-use risk and fairness obligations that we state plainly. *Misuse.* The same attention weights that make a decision auditable also reveal which feature patterns the detector keys on, which an adversary could in principle use to shape evasive behavior; the adversarial evaluation of Section 6.10 is included precisely to quantify how much that helps an attacker, and we recommend that deployed instances keep the per-decision explanations on the infrastructure side rather than broadcasting them. A trust score must also never be repurposed as a long-term reputation or tracking signal for individual drivers; it is a transient, per-window safety signal and should be discarded after use, consistent with data-minimization principles. *Fairness.* Because the features are kinematic and protocol-level rather than identity-linked, the detector does not use any attribute tied to a driver or vehicle owner. It can, however, behave unevenly across operating conditions: vehicles with unusual but legitimate mobility (emergency vehicles, heavy goods vehicles that accelerate slowly, vehicles in dense stop-and-go traffic) sit closer to the decision boundary and risk higher false-positive rates. The online tuning of AFMT is intended to track such regime shifts, but a safety-critical deployment should monitor per-class false-positive rates across vehicle types and traffic densities and calibrate the threshold per deployment rather than assume a single global operating point.

### 7.3. Limitations and Future Work

We note the remaining limitations plainly: (i) The detector is supervised and requires labeled attack traces; semi-supervised adaptation to unlabeled in-the-wild data is left to future work. (ii) The adversarial evaluation uses feature-level PGD; model-stealing and membership-inference attacks are out of scope. (iii) We focus on position-based misbehavior; multi-vehicle Sybil coalitions are not addressed. (iv) The primary dataset is simulator-synthesized, and the real-data validation covers only the kinematic feature subset; a real-trace deployment that records NDN-plane features is the natural next step. Future work will pursue that deployment together with semi-supervised pretraining and self-distilled fuzzy rules. Scaling the evaluation to denser fleets and collision-aware scenarios would also benefit from high-fidelity parallel vehicular simulators such as the bounding-volume-hierarchy collision simulator of Grinberg and Wiseman [33], which could drive larger and more realistic multi-vehicle attack scenarios than the present testbed.

## 8. Conclusions

We presented a two-stage misbehavior detector for Vehicular NDN that combines an online-tuned fuzzy pre-filter (AFMT) with an attention-augmented bidirectional LSTM. The fuzzy stage stays informative because its membership-function parameters are tuned online, in the same computational graph, by the BiLSTM’s prediction error; the attention stage stays interpretable because it emits per-time-step weights aligned with attack onsets. On a simulator-synthesized VNDN benchmark spanning the five canonical attack types, the detector reaches F1 between 0.955 and 0.979 (macro-average 0.964), ties the strongest baselines on the hardest attack while attaining the highest ROC-AUC, retains 99.2% of its F1 under an internal distribution shift, and runs in 0.44 ms per sample on a CPU. On a live OMNeT++/Veins/SUMO testbed built on our published VeReMiVNDN platform [17] (the LuST (Luxembourg) scenario), it attains F1 0.986. On the real public VeReMi v1.0 dataset it transfers to four of the five attack types at F1 near 1.0, with Constant Offset remaining invisible to kinematics-only features, which quantifies the value of the named-data-plane features that a real deployment should record. Future work will pursue a real-trace deployment that captures those features on a campus VNDN testbed, together with semi-supervised pretraining and self-distilled fuzzy rules.

## Figures and Tables

**Table 1:** Position of the proposed detector relative to representative recent VNDN/VANET misbehavior detectors. Column legend (AF, AT/XAI, AR, PA-F1, Lat.) is defined in the text; “✓” present, “–” absent.

Work/Year	Dataset	AF	AT/XAI	AR	PA-F1	Lat.
Khan & Lim, 2022 [12]	VANET-PoC	–	–	–	–	–
Siddiqa et al., 2021 [18]	Hand-crafted	–	–	–	–	–
Jadhav et al., 2023 [6]	VeReMi	–	–	–	✓	–
Alashjaee et al., 2025 [7]	Generic NIDS	–	✓	–	–	–
AttentionGuard, 2025 [8]	Platoon traces	–	✓	–	–	–
Dual-Branch T-BiLSTM, 2025 [9]	VeReMi	–	✓	–	✓	–
Sharma & Jaekel, 2022 [21]	VeReMi	–	–	–	✓	–
VeMisNet, 2025 [20]	VeReMi	–	–	–	–	✓
Proposed (AFMT + Att-BiLSTM)	Synth. + real VeReMi	✓	✓	✓	✓	✓

**Table 2:** Configuration of the five VeReMi attack types used in the evaluation, with the per-beacon manipulation, the underlying attacker intent, and the detection-side difficulty observed empirically.

Type	Name	Per-Beacon Manipulation	Attacker Intent	Difficulty
1	Constant Position	Transmit the same fixed (x,y) in every beacon.	Hide location.	Low
2	Constant Offset	Shift reported (x,y) by a fixed bias vector.	Cause small-scale corruption of trust state.	Low
4	Random Position	Uniformly sampled (x,y) within a 4 km square.	Generate ghost vehicles.	Medium
8	Random Offset	Per-beacon bias drawn uniformly in [−15,15] m.	Evade threshold-based detectors.	High
16	Eventual Stop	Normal behavior until a random instant, then position freezes.	Disguise traffic obstruction.	Medium

**Table 3:** Notation used throughout this paper, grouped by data-flow stage.

Symbol	Unit/Range	Meaning
*N*	vehicles	Fleet size in the simulation.
α	∈[0,1]	Attacker density (fraction of malicious vehicles).
*W*	packets	Sliding observation window, *W* = 20.
*T*	time-steps	BiLSTM sequence length per detection decision, *T* = 60.
$x_{v}^{\left(t\right)}$	$R^{5}$	5-D behavioral feature vector of vehicle *v* at packet index *t*.
*d*	dimensionless	Feature dimension, *d* = 5 (raw) or *d* = 6 (with FS).
*h*	dimensionless	BiLSTM hidden size per direction, *h* = 128.
$\mu_{j,\ell }\left(x;a,b,c\right)$	∈[0,1]	Triangular MF for feature *j* and linguistic label *ℓ*, constrained to a≤b≤c.
$z_{\ell }$	∈{0.1,0.5,0.9}	Fixed consequent of rule *ℓ*.
$w_{j}$	>0	Softplus-gated per-feature risk weight.
FS(v)	∈[0,1]	AFMT fuzzy suspicion score for vehicle *v*.
${\hat{y}}_{v}$	∈[0,1]	BiLSTM-predicted attacker probability for *v*.
$\tau \left(v\right)=1-{\hat{y}}_{v}$	∈[0,1]	Trust score reported by the detector.
$\alpha_{t}$ (attention)	∈[0,1]	Attention weight at time-step *t* (per-decision explanation).
$\eta_{t}$ (AFMT learning rate)	$R^{+}$	Step size used in the projected gradient update of MFs.
ϵ	$\ell_{\infty }$	PGD perturbation budget of the adaptive adversary.
λ	$R^{+}$	Weight of the AFMT auxiliary loss, λ = 0.20.
γ	$R^{+}$	Focal-loss focusing parameter, γ = 2.

**Table 4:** Model size of the proposed detector and the baselines. Parameter counts are exact; size is at float32.

Model	Parameters	Size (MB)
Consecutive-BSM ML	18,177	0.07
GRU (2-layer)	151,041	0.58
CNN-LSTM	244,929	0.93
Transformer (3L, 4-head)	398,337	1.52
LSTM (4-layer)	465,537	1.78
BiLSTM (2-layer)	533,761	2.04
Proposed (AFMT + Att-BiLSTM)	930,867	**3.55**

**Table 5:** Parameters of the OMNeT++/Veins/SUMO VNDN testbed (and of the matched synthesized dataset). The radio, PHY, and mobility settings follow the IEEE 802.11p profile used by the VeReMi authors so that detection numbers are comparable to prior work.

Parameter	Value
Reference network stack	OMNeT++ 6.0.3 + Veins 5.3 + INET 4.5
Mobility model	SUMO 1.20, LuST (Luxembourg) scenario
Map area	LuST road network (≈15×16 km playground)
Fleet size *N*	50, 100, 200, 500, 1000 vehicles
RSU spacing	500 m
Radio	IEEE 802.11p, 5.9 GHz center, 10 MHz bandwidth
PHY	OFDM; log-distance path loss + Nakagami-*m* (*m* = 3), $\sigma_{\mathrm{shadow}}=4$ dB
Transmit power	20 dBm (effective range ≈300 m)
Beacon (BSM) interval	100 ms
Interest/data lifetime	1 s/10 s
Modeled scenario duration	600 s, with a 30 s warm-up phase
Dataset generation	deterministic per (attack type, density); fixed master seed

**Table 6:** Representative window-averaged feature vectors (range [0,1]) for a benign and an attacking vehicle, from the simulator-synthesized Type 8 set and from the real VeReMi v1.0 kinematic set. For the synthetic rows the columns are $f_{1}$ to $f_{5}$; for the real rows they are the kinematic counterparts $g_{1}$ to $g_{5}$ defined in Section 5.2.

Source	Class	(1)	(2)	(3)	(4)	(5)
Synthetic (T8)	benign	0.178	0.344	0.193	0.503	0.382
Synthetic (T8)	attacker	0.666	0.433	0.164	0.378	0.404
Real VeReMi	benign	0.493	0.008	0.022	0.005	0.108
Real VeReMi	attacker	0.332	0.283	0.018	0.005	0.000

**Table 7:** Training hyperparameters used to train the proposed detector and the six deep learning baselines on identical splits. All hyperparameters are fixed across attack types and fleet sizes; only the final decision threshold is retuned per validation split.

Parameter	Value
Optimizer/learning rate	Adam, $\eta_{0}=10^{-3}$ with cosine annealing to $10^{-5}$
Weight decay	$10^{-5}$
Batch size	128
Epochs (early-stop patience)	28 (patience 6 on validation F1)
BiLSTM hidden *h*/layers/dropout	128/2/0.3
Attention head count	4-head self-attention pool with a learnable query
Loss function	Focal BCE (γ = 2, positive-class weight 1.8) + $\lambda \;L_{\mathrm{AFMT}}$, λ = 0.20
AFMT MF projection	Once per minibatch; enforces a≤b≤c
Detection-threshold tuning	Grid over [0.02,0.98], step 0.01; max F1 on validation
Adversarial training (robustness)	Multi-step PGD, $\epsilon_{\mathrm{train}}=0.12$, 3 steps, step-size ϵ/3
Random seeds	{1,2,3}; best-of-3 on validation F1 reported
Implementation	PyTorch 2.11; CUDA 12.3; NVIDIA RTX 3090 (24 GB)

**Table 8:** Per-attack-type detection performance at attacker density α=0.3. Numbers are measured on a held-out test split (best of three seeds on validation F1).

Attack Type	Accuracy	Precision	Recall	F1	ROC-AUC	MCC
Constant (T1)	0.9756	0.9565	0.9635	0.9600	0.9837	0.9424
Constant Offset (T2)	0.9756	0.9850	0.9357	0.9597	0.9645	0.9428
Random (T4)	0.9800	0.9848	0.9489	0.9665	0.9728	0.9526
Random Offset (T8)	0.9756	0.9590	0.9512	0.9551	0.9842	0.9383
Eventual Stop (T16)	0.9867	0.9856	0.9716	0.9786	0.9855	0.9689
**Macro-average**	**0.9787**	**0.9742**	**0.9542**	**0.9640**	**0.9781**	**0.9490**

**Table 9:** Comparison against six baselines at α=0.3 on Type 8 (Random Offset). Latency is per-sample CPU inference. Bold = proposed.

Model	Accuracy	Precision	Recall	F1	ROC-AUC	CPU Lat. (ms)
LSTM (4-layer)	0.9667	0.9426	0.9350	0.9388	0.9731	0.164
BiLSTM (2-layer)	0.9578	0.9194	0.9268	0.9231	0.9777	0.203
GRU (2-layer)	0.9756	0.9590	0.9512	0.9551	0.9786	0.125
CNN-LSTM	0.9778	0.9748	0.9431	0.9587	0.9737	0.088
Transformer (3L, 4-head)	0.9600	0.9268	0.9268	0.9268	0.9786	0.140
Consecutive-BSM ML [21]	0.9711	0.9435	0.9512	0.9474	0.9815	0.010
**Proposed (AFMT + Att-BiLSTM)**	**0.9756**	**0.9590**	**0.9512**	**0.9551**	**0.9842**	**0.439**

**Table 10:** Subtractive ablation on Type 8 at α=0.3. Removing the attention pool costs 1.73 F1 points and removing AFMT costs 0.47.

Configuration	Accuracy	F1	ROC-AUC	MCC
Full (AFMT + Att-BiLSTM)	0.9756	0.9551	0.9842	**0.9383**
w/o attention (mean pool)	0.9667	0.9378	0.9788	0.9154
w/o AFMT (no fuzzy pre-filter)	0.9733	0.9504	0.9808	0.9324
w/o focal loss (plain BCE)	0.9778	0.9590	0.9803	0.9438
w/o fuzzy auxiliary loss (λ = 0)	0.9778	0.9590	0.9799	0.9438
ΔF1 (full − w/o attention) =+1.73; ΔF1 (full − w/o AFMT) =+0.47 (points).				

**Table 11:** Incremental ablation: components added to the plain BiLSTM baseline (Type 8, α=0.3).

Configuration	Accuracy	F1	ROC-AUC	MCC
Baseline BiLSTM (mean pool)	0.9578	0.9231	0.9777	0.8940
+ attention pool	0.9778	0.9590	0.9817	0.9438
+ AFMT fuzzy pre-filter	0.9778	0.9590	0.9803	0.9438
+ focal loss (full)	0.9756	0.9551	0.9842	0.9383

**Table 12:** Per-feature importance by leave-one-feature-out retrain (Type 8, α=0.3). ΔF1 is the drop relative to the full five-feature model (F1 = 0.9551).

Removed Feature	F1 (4 Features)	ΔF1
$f_{1}$ TTL variability	0.7281	0.2270
$f_{3}$ packet-drop rate	0.9286	0.0265
$f_{5}$ Interest forwarding rate	0.9469	0.0082
$f_{2}$ Interest-to-data ratio	0.9547	0.0004
$f_{4}$ position plausibility (Kalman)	0.9547	0.0004

**Table 13:** AFMT design choices: the membership-function shape shape and rule-count study (Type 8, α=0.3).

AFMT Configuration	Accuracy	F1	ROC-AUC
Triangular, 3 sets (default)	0.9778	0.9590	0.9800
Gaussian, 3 sets	0.9778	0.9590	0.9798
Trapezoidal, 3 sets	0.9778	0.9590	0.9798
Triangular, 2 sets	0.9756	0.9547	0.9800
Triangular, 5 sets	0.9778	0.9590	0.9800

**Table 14:** Scalability across fleet sizes from 50 to 1000 vehicles (Type 8, α=0.3).

*N* (Vehicles)	Accuracy	Precision	Recall	F1
50	0.9633	0.9560	0.9255	0.9405
100	0.9633	0.9560	0.9255	0.9405
200	0.9633	0.9560	0.9255	0.9405
500	0.9633	0.9560	0.9255	0.9405
1000	0.9600	0.9602	0.9086	0.9337

**Table 15:** Adversarial robustness under PGD-$\ell_{\infty }$ evasion on Type 8 at α=0.3 ($\epsilon_{\mathrm{train}}=0.12$). Budgets beyond ϵ≈0.04 exceed realistic sensor noise.

ϵ ($\ell_{\infty }$)	Accuracy	Precision	Recall	F1
0.00	0.9711	0.9583	0.9350	0.9465
0.02	0.9467	0.8960	0.9106	0.9032
0.04	0.9000	0.7671	0.9106	0.8327
0.06	0.8022	0.5914	0.8943	0.7120
0.08	0.6622	0.4372	0.8211	0.5706
0.10	0.4733	0.3137	0.7805	0.4476
0.12	0.2733	0.2302	0.7073	0.3473

**Table 16:** Real public VeReMi v1.0 external validation on the kinematic feature subset (2124 real vehicle sequences, 445 attackers). F1 is near 1.0 on four attack types; Constant Offset is invisible to kinematics-only features (see text).

Regime	Accuracy	Precision	Recall	F1	ROC-AUC
Overall (real VeReMi)	0.9375	0.9792	0.7121	0.8246	0.8331
Constant (T1)	1.0000	1.0000	1.0000	1.0000	1.0000
Constant Offset (T2)	0.7778	0.0000	0.0000	0.0000	0.4233
Random (T4)	1.0000	1.0000	1.0000	1.0000	1.0000
Random Offset (T8)	1.0000	1.0000	1.0000	1.0000	1.0000
Eventual Stop (T16)	1.0000	1.0000	1.0000	1.0000	1.0000

**Table 17:** Validation on the live OMNeT++/Veins/SUMO testbed (LuST scenario, 780 real vehicle sequences, 194 attackers). Metrics are on a held-out test split; features are logged directly from the running NDN stack.

Regime	Accuracy	Precision	Recall	F1	ROC-AUC	MCC
OMNeT++/Veins/SUMO (LuST)	0.9915	0.9714	1.0000	0.9855	1.0000	0.9797

**Table 18:** Robustness to an internal distribution shift (default profile versus a shifted profile of the same simulator). This is a within-simulator stress test; see Section 6.11 for real-data validation.

Regime	Accuracy	Precision	Recall	F1	ROC-AUC	MCC
default profile	0.9756	0.9590	0.9512	0.9551	0.9842	0.9383
shifted profile	0.9673	0.9658	0.9289	0.9470	0.9631	0.9237
Macro-F1 retention under shift: 0.9470/0.9551=0.992.						

## Data Availability

The real public VeReMi v1.0 dataset used for external validation is openly available from its maintainers (https://veremi-dataset.github.io/) (accessed on 23 June 2026). The OMNeT++/Veins/SUMO testbed extends the VeReMiVNDN platform [17]. The testbed extension (attack modules and feature collector), the simulator-synthesized VNDN dataset, the trained PyTorch checkpoints, and the data-generation and figure scripts used in this study are available from the corresponding author on reasonable request.

## Abbreviations

The following abbreviations are used in this manuscript:

AFMT	Adaptive Fuzzy Membership Tuning
BiLSTM	Bidirectional Long Short-Term Memory
BSM	Basic Safety Message
CNN	Convolutional Neural Network
CS	Content Store
FIB	Forwarding Information Base
GRU	Gated Recurrent Unit
IDS	Intrusion Detection System
LSTM	Long Short-Term Memory
MCC	Matthews Correlation Coefficient
NDN	Named Data Networking
OBU	On-Board Unit
PDR	Packet Drop Rate
PGD	Projected Gradient Descent
PIT	Pending Interest Table
RSU	Road-Side Unit
SUMO	Simulation of Urban MObility
VANET	Vehicular Ad Hoc Network
VNDN	Vehicular Named Data Networking
